# PP33 A Mathematical Model For The Clinical Assessment Of Mechanical Ventilators

**DOI:** 10.1017/S0266462325102523

**Published:** 2025-12-29

**Authors:** K. Paola López-López, Ana B. Pimentel-Aguilar, Martha R. Ortiz-Posadas

## Abstract

**Introduction:**

Clinical assessment is a continuous process during the life cycle of a medical device for obtaining evidence about performance, safety, demand, and effectiveness in patient care. The objective of this work was to carry out the clinical evaluation of a sample of 66 mechanical ventilators from the National Institute of Respiratory Diseases in Mexico.

**Methods:**

The multi-criteria decision analysis method was used. Eleven variables were defined: three related to the patient (number of patients treated, illness, and average length of stay), and eight related to the medical device (amount, location, type, function, use hours, out of service hours, brand, and model). Three partial indicators were developed to evaluate availability, demand, and operation aspects of the medical device. These partial indicators were integrated into a global indicator called clinical use. The numerical result was interpreted with a qualitative scale defined with four levels of clinical use (low, regular, medium, and high). Data from the year 2023 were used.

**Results:**

The sample evaluated contained seven brands and 12 different models of mechanical ventilators. Forty-two percent were classified as low clinical use (they are underutilized) and 39 percent were regular clinical use. These two results indicated that these 54 devices have a low impact on patient care. On the other hand, only 11 percent had medium use and 8 percent had high use. In this last case, we must note the overuse of these five ventilators; that is, they were used almost 24 hours a day in patient care.

**Conclusions:**

Sixty-two devices in the sample had a clinical use that allowed them to be available for mechanical ventilation of patients with respiratory conditions that require it. In the case of ventilators with high clinical use, technological improvements due to the brand had an influence on their constant use, allowing effective care for the patients in critical condition.

